# *Brokered dialogue*: A new research method for controversial health and social issues

**DOI:** 10.1186/1471-2288-12-92

**Published:** 2012-07-02

**Authors:** Janet A Parsons, James V Lavery

**Affiliations:** 1 Applied Health Research Centre, Li Ka Shing Knowledge Institute, St. Michael’s Hospital, Toronto, Canada; 2 Centre for Research on Inner City Health and Centre for Global Health Research, Li Ka Shing Knowledge Institute, St. Michael’s Hospital, Toronto, Canada; 3 Department of Physical Therapy, University of Toronto, Toronto, Canada; 4 Dalla Lana School of Public Health and Joint Centre for Bioethics, University of Toronto, Toronto, Canada

## Abstract

**Abstract:**

**Background:**

Dialogue is a foundational feature of social life and an important way in which we come to understand one another. In situations of controversy dialogue is often absent because of a range of social barriers. We have developed a new film-based qualitative research method for studying controversial issues in healthcare and social policy. We call this method *Brokered Dialogue*. Theoretically informed by the traditions in narrative inquiry and visual anthropology, the method is premised on the idea that dialogue possesses features making it unique as a generator of new knowledge and opportunities for social intervention. Film is not only an extraordinarily rich data source, but an excellent medium for knowledge transfer and dissemination.

**Discussion:**

The paper introduces the *Brokered Dialogue* method. We outline its critical steps, including the procedures for sampling, data collection and data analysis of both textual and visual data. Participants in a *Brokered Dialogue* engage in filmed interviews that capture their perspectives on a given topic; they then share their perspectives with, and pose questions of, one another through the medium of film. Using a participatory editing process, only footage that participants feel comfortable showing to others is incorporated. This technique offers participants a ‘safe’ space for respectful interaction. The editing process itself is analytic, and the final assembly of footage approximates a dialogue on the topic at hand. A link to a film produced from a project piloting the method is provided to demonstrate its real world application.

**Summary:**

*Brokered Dialogue* is a method for promoting respectful interactions among those with seemingly divergent views on a controversial topic and for discovering critical points of divergence that may represent pathways for improvement. While the end product is a ‘film’, the goal is to have these films used as catalysts for ongoing respectful dialogue and problem-solving concerning the topic at hand informing relevant practice and policy change. In this paper, we consider *Brokered Dialogue*’s potential future uses and impacts, and how these might be evaluated.

## Background

Dialogue, which means literally “through words”, is a foundational feature of social and political life, and one that we often take for granted. Dialogue reveals individuals’ fundamental values and interests. It is how we come to know one another. It is also an important feature of deliberative democracy. It is through dialogue that we confront and accommodate diverse interests in a range of public contexts.

Dialogue entails conversation, but it is also a performance [1,2]. We portray ourselves to others when we interact, both verbally and non-verbally, and we interpret their portrayals of themselves in turn. How we understand the portrayals of others and how they understand our own is vital to how the dialogue unfolds, what is communicated, how it is interpreted, the tone of the interaction, and our impressions of the other person. As such, it can entail many auditory, visual and embodied cues for both teller and listener [3].

Dialogue offers enormous potential as a research tool. Analyzing dialogue can reveal critical points of disagreement, as well as opportunities for resolution and reconciliation in socially controversial issues.

In healthcare, when communication breaks down and fault lines arise between and among affected parties, the consequences may be substantial. This occurs frequently across the spectrum, from research and clinical practice contexts to complex issues in health policy and bioethics. But despite the pervasive rhetoric about the importance of good communication as a foundational principle of quality healthcare, anecdotal evidence abounds about communication breakdowns throughout the healthcare enterprise, and about the lack of opportunities for authentic dialogue among affected parties. For example, the clinical encounter is a situation where dialogue may be impeded for a host of reasons, including social structural factors, and differences in understanding of disease processes between patient and clinician [4]. Both parties approach the encounter informed by their own contexts and assumptions. Another example is in the field of knowledge translation: initiatives aimed at changing clinicians’ practice behaviours sometimes meet with limited or no success, precisely because no one asks the end-users why they behave in the ways they do. Both practitioners and patients frequently do not do what they are ‘supposed’ to do for very informed and logical reasons that are consistent with their own perspectives and contexts. Dialogue provides opportunities for communicating underlying assumptions, sharing lived experiences, and fostering understanding of the perspectives of others.

In this paper we describe a new method called *Brokered Dialogue* which we have developed to understand and address absences of dialogue in healthcare and other social settings. It is designed to study controversial situations where the lack of opportunities for dialogue has given rise to significant disagreement, or has been an obstacle to progress, or has been an impediment to developing constructive relationships. In this paper, we describe the methodological features of *Brokered Dialogue*.

In separate publications we will describe the application of the *Brokered Dialogue* method in various contexts.

*Brokered Dialogue* is premised on the idea that there are features of dialogue that make it unique as a generator of new knowledge and opportunities for social intervention:

1) Dialogue can isolate and help to clarify the nature and scope of disagreements and differences of perspective and values among interested parties involved in an issue.

2) Dialogue can reveal where positions/perspectives may be susceptible to change/revision/refinement. This is likely most important in cases where the assumptions underlying the various positions seem most polarized or entrenched (‘hardened’).

3) Dialogue can concretize and contextualize abstract concepts such as ‘fairness’ and ‘justice’ and make these ideas meaningful for stakeholders confronted with complex social challenges in their everyday experience [5].

4) Dialogue can illuminate opportunities for reconciling divergent perspectives.

5) Dialogue can illuminate pathways to potentially effective solutions or resolutions to issues for which there is entrenched disagreement among the interested parties.

### Barriers to dialogue

There are a host of reasons why dialogue about controversial issues in healthcare and other contexts may be difficult, or not occur at all. Many of these are interrelated. One barrier is the *lack of appropriate contexts* for dialogue. For example, the structure of fee-for-service medical practice may present a barrier to meaningful dialogue between physicians and patients. With only minutes available to interact with each patient within the demands of a busy clinical practice, the free exchange of perspectives and interests may appear impossible to both patient and clinician. For example, Britten and colleagues identified that physicians assumed patients presenting with cold symptoms would settle for nothing less than an antibiotic prescription, while patients did not want to appear to contradict or burden their ‘busy’ doctors even though they said they did not see the antibiotic as effective treatment [6]. The assumptions were unspoken between both parties and remained hidden until Britten asked each group of participants to explain their behaviour and assumptions. Arthur Frank has eloquently described the potential benefits of dialogue within the clinical encounter [4]. He argues that the inability to enter into meaningful dialogue is ‘de-moralizing’ for both patient and physician [4]. Conversely, when genuine dialogue happens, patients feel empowered and engaged in their own care, and physicians are able to treat their patients as whole people, more than ‘just’ their diagnoses [4]. This sense of partnership, fostered by dialogue, makes for a more mutually satisfying experience for clinician and patient [4].

Another barrier to dialogue may arise from *disparities in education and/or language skill*. Lay people may feel unqualified to comment on health policy matters and may be content to defer to perceived experts, despite what may be important stakes for them in how these matters are managed. These attitudes fuel power imbalances, which determine whose perspectives are taken into consideration in crafting policy solutions. Frank [4] and others [2] have argued that the perspectives of biomedicine have traditionally been privileged in considerations of disease and illness, and it is only comparatively recently, and still only in certain circles, that lay and patient perspectives have begun to be valued.

Another critical barrier to dialogue in healthcare occurs as a result of stigma and marginalization. For example, a homeless person may be reluctant to enter into dialogue with an affluent health professional, because of the personal trauma experienced over time by ‘damaging stories’ about homeless people [7,8]. This experience of a ‘spoiled’ identity was central to Goffman’s initial conceptualization of stigma, which emphasized that stigmatized individuals feel that others do not really accept them and are not ready to make contact with them on equal grounds [1,9].

Lack of dialogue can perpetuate the avoidance of others perceived to be different, or having views counter to their own. An extreme example of this is seen in ‘gated communities’, which drastically reduce the likelihood of social interactions between affluent and non-affluent citizens of a region, and effectively foreclose any dialogue in which they might otherwise engage. The result is a loss of experience with different perspectives, which can contribute to an insular world view, on the one hand, and experiences of social exclusion and devaluation on the other [10].

### The positive power of dialogue

The *Brokered Dialogue* method is premised on the idea that there is something unique about dialogue as a generator of new knowledge [11]. We hypothesized that if we could identify important issues in which barriers to communication had arisen, and if we could encourage interested parties to engage in dialogue with one another—especially when that dialogue would likely not otherwise have occurred—then opportunities for new insights and increased understanding would result. By providing a hub to link people with divergent perspectives, the *Brokered Dialogue* method can also serve as a social intervention, potentially contributing to the resolution of disagreements through a variety of applications, discussed in greater detail below. This aspect of *Brokered Dialogue* draws on the narrative theories of Levinas and Ricoeur, which emphasize that it is through stories and dialogue that we come to recognize ‘oneself in another’ [12,13]. As such, dialogue may foster recognition of similarities between our own perspective and those of others, which may help to temper the emphasis on differences that often occur in controversial issues, and open new avenues for progress.

The stories we tell in sharing our perspectives serve both descriptive and prescriptive functions [13]. We articulate not only our ‘take’ on the problem, but we also offer suggestions for, or directions towards, what we believe ‘ought’ to happen. The sharing of stories and perspectives does not stop with the individual perspective, but the power rests in how such stories are taken up by others, interpreted, responded to, and incorporated into their own perspectives [4,14]. Underlying many controversies is a perspectival distancing between parties with ‘opposing’ views. Drawing on Trede’s conceptualization of critical transformative dialogues, parties engaged in dialogue may discover the opportunity to explore beyond their own horizons [15]. Dialogue reveals, and confronts the participants with, the values, power relationships and full range of personal stakes in a given issue and thereby bridges the personal and public spheres.

*Brokered Dialogue* is a method for capturing a representative range of interests and perspectives on a controversial issue, promoting respectful interactions among those with seemingly divergent views, and identifying potential pathways through which the issue might be solved or improved. We view the act of *recognizing* the interests and perspectives of ‘others’ in a polarized debate as a critical first step towards resolving disagreements which can present obstacles to progress. Recognizing points of agreement serves to legitimize the perspectives of others, as well as one’s own, and provides opportunities for bridging the distance between self and others [12,13]. Trede describes this process of mutual recognition as a ‘fusion of horizons’, or the ability to see, and possibly share, the ‘horizons’ of others. It is here where meaningful dialogue becomes possible. It is also the ability to engage freely in critical reflection with one’s own and others’ perspectives that is potentially transformative [15].

The virtual space for interaction created through the sharing of film clips that is central to the *Brokered Dialogue* method is both literally and figuratively a ‘fusion of horizons’ into a ‘safe’ space for a respectful and thoughtful dialogue.

### Why film? accessibility, representation and audience

Film is the main vehicle by which the *Brokered Dialogue* method converges the perspectives at play in any controversial issue. We use it both as a research tool in its own right, and as a means for public engagement, social intervention and knowledge translation. Film is an accessible and familiar medium, which makes it a particularly useful vehicle for public engagement on complex social issues [16-18]. Beyond this however, it provides a much richer data source than purely text-based or even audio-based data. Our method is informed by the long tradition within anthropology and sociology of using film to record observations, interviews and interactions; to illuminate similarities and differences between cultural groups; and to engage viewers [16,18-20]. Using a visual medium like film situates the method within an arts-based approach to research that attempts to emphasize interconnectedness and relational aspects of experience, bringing affective and cognitive understanding together [21].

There is also a theoretical rationale for including film. Levinas and Ricoeur argued for narrative’s—and dialogue’s–important role in allowing one to recognize another person, of seeing ‘oneself in another’ while acknowledging their sovereignty or separateness from us [12,13]. Levinas used the concept of ‘face’ to describe this process [12]. Seeing the faces of others is important for recognizing our common humanity and for fostering obligations to listen and respond to other persons respectfully [22]. In order to offer participants an opportunity to engage with and reflect upon the perspectives of others, and to articulate their own perspectives with care and respect, the participants in our *Brokered Dialogues* do not meet face to face. *Brokered Dialogue* employs a participant-driven editing process, described below, which allows participants in the dialogue to represent their perspectives on a topic in a way that they feel comfortable sharing with other people. Meeting face-to-face might be uncomfortable or awkward, especially in the context of controversies or social situations in which there are power imbalances among the interested parties, such as a homeless patient questioning an emergency room physician’s attitude towards her. The use of film, with its highly accessible editing capabilities, provides a ‘safe’ and exploratory space for sharing, and being confronted by, the stories and perspectives of others.

Film is also an important medium for representation. Representation of an issue and representation of key stakeholders’ perspectives can occur seamlessly and efficiently on film. As Michael Bérubé comments, “representations matter” [23]. They shape the experiences of those who are represented (or misrepresented), how the various stakeholders understand an issue, and how they interpret the accounts of others. Through the editing process, participants in a *Brokered Dialogue* are not required to share any footage or commentary that they feel would either misrepresent their position on a topic, or that might cast them ‘in a bad light’. As researchers, we track participants’ editing choices and how they respond to their own ‘raw’ footage, what they choose to include and exclude. The material that is not included in a finished ‘cut’ of the resulting film(s) can still be used in traditional qualitative analysis, providing us with useful insights about how concerns about representation might contribute to the dynamics of a given issue, including insights about obstacles to dialogue itself. There is a democratizing impulse therefore to the method, in that participants and researchers both play a role in shaping the meta-narrative of the resulting film, which approximates a dialogue among the stakeholders.

### ‘Creative applications’

There may be instances where a film is not the most appropriate end product for widespread public dissemination of research findings resulting from a given *Brokered Dialogue* study. In cases of severe stigmatization for example, where the identification of research participants might result in important and potentially detrimental consequences for them, we may need to use a variety of ‘creative applications’ that remain true to the study findings, but disseminate them in another equally engaging format. For example, instead of a film, actors could be used to produce re-enactments of the interview data, in order to protect the identity of participants. Animation might be another option for such creative applications. We intend to explore these options as appropriate in future projects.

### Use and impact

Ideally, the resulting films produced via *Brokered Dialogue* should be rich conceptually, and should clearly reflect the four goals of analysis: 1) they should reveal an in-depth understanding of the positions from which participants begin the dialogue; 2) they should demonstrate what the major points of contention are or where the relevant perspectives diverge significantly regarding the topic at hand; 3) they should consider whether the participants’ perspectives changed considerably by engaging in the *Brokered Dialogue*; and 4) hopefully, they will propose novel solutions or pathways towards resolution or at least promote greater understanding of the perspectives between the ‘players’. The real power of the method lies not in the creation of the film itself, but in using the films as catalysts for further dialogue and promoting greater understanding out in the ‘real world’ beyond the dialogic circle under study. As such, the film once distributed/disseminated is not a conclusion but rather a beginning, resulting in an ongoing multiplier effect as different audiences engage with it, take it up and participate in further dialogue and practice/policy change. We see it as a tool for facilitating social innovation, one that is informative to the fields of not only health practice and policy, but also knowledge translation, conflict resolution, change management, and community and public engagement.

In terms of measuring potential ‘impacts’ of the films (and additional creative applications), we are currently devising methods for identifying the metrics to evaluate such impacts. Relatively few evaluation strategies exist for assessing impacts of arts-based research methods, and virtually none involving research-based film [22,24]. The extent to which it changes viewers’ perceptions on the topic, whether it increases understanding between those with divergent perspectives, and whether the solutions identified are recognized by viewers are all potential features of change we would be interested in assessing [2]. For example, interactive survey technologies could be used prior to a screening of the film and then immediately afterwards to gauge shifts in opinion on particular features of the controversy. These strategies could be complemented with in-depth interviews or focus groups, to gain a better understanding of the impact of the *Brokered Dialogue* method on attitudes, opinions and on behavior changes. Repeated measures over time might help us understand the temporal duration of any impact. Future studies will build an evaluation framework.

## Discussion

Table 1 summarizes the critical steps involved in the *Brokered Dialogue* method.

**Table 1:** **Critical Steps in*Brokered Dialogue***

***Step No.***	***Critical Step***	***Description***
**1**	**Issue/topic selection**	**General requirements:**
		**a) issue of importance/social significance**
		**b) lack of publicly accessible dialogue among stakeholders, contributing to, or resulting in some form of challenge, controversy, problem or obstacle.**
**2**	**Introductory filmed interviews (documenting perspectives)**	**- in-depth open-ended filmed interviews conducted with each participant**
		**- Purposive sampling strategy: looking for a range of perspectives on a topic**
		**- Documentation of “baseline” positions to permit analysis of changes in positions/perspectives as a result of participation in the*Brokered Dialogue***
**3**	**Identification of preliminary dialogue domains**	**- combined approach of reviewing initial filmed interviews and written transcripts (open coding)**
		**- identification of emergent themes and patterns within and between participants’ accounts (constant comparison)**
**4**	**‘First cut’ edits**	- **based on initial analysis, first ‘cuts’ of individual interviews assembled**
		**- resultant film clips highlight key points of individual participants’ perspectives on topic**
		**- participatory editing process ensures participants are comfortable showing clips to other participants (decisions recorded)**
**5**	**Responses to first cuts**	**- first cut clips shared with other participants and responses filmed**
**6**	**Analysis of responsive sessions and ‘second cut’ edits**	- **building on initial analysis, review of responses and written transcripts (as per step 3 above)**
		- **focus oninteractionwith perspectives of others**
		- **results in a ‘second cut’ of footage from responses (as per step 4 above – including participatory editing)**
**7**	**Repeat**	- **The number of iterations depends on the complexity of the issue and dialogue, and the feasibility of continued iterations**
**8**	**Rough cut and assembly**	- **review of all approved footage, related transcripts by research team**
		**- iterative analysis between filmed footage and transcripts (constant comparison)**
		**- final decisions regarding inclusion of perspective-sharing/response/rebuttal (selective coding)**
		**- interview segments assembled to approximate dialogue**
		**- Aim is:**
		**- (a) present unique insights arising from the dialogue**
		**- (b) fair representation of the normative structure of the dialogue**
		**- (c) reflect any evolution or change of perspective for individual participants that have become apparent over the course of the*Brokered Dialogue***
**8**	**Revisions and approval**	**- Rough cut assembly viewed by each participant**
		**- participants provide editorial feedback, can request edits to own footage (edits incorporated)**
		**- Each participant must provide approval for use of their footage**
**9**	**Final Cut and post-production**	**-Final versions of the film are prepared.**
		**-The length and content can be tailored to specific uses, e.g., teaching vs. a presentation at a public forum**

The method begins with the selection of a socially significant issue or controversy in which there is a lack of dialogue among the relevant stakeholders. Participants are selected based on the particular perspective they are anticipated to bring to the dialogue (purposive sampling) [25]. For example, we might conduct a *Brokered Dialogue* about seasonal influenza vaccination among healthcare workers, since vaccination rates remain low worldwide despite explicit recommendations that they be vaccinated [26]. We might begin by interviewing frontline practitioners who had and had not been vaccinated and perhaps an occupational health clinician involved in promoting staff vaccination and surveillance at a given institution.

Once participants have been identified, in-depth open-ended filmed interviews are conducted with each participant in order to elicit their experiences and perspectives on the topic. Drawing on interactive narrative methods, the positionality or ‘footing’ of given participants is recognized as important [27]. Based on our preliminary thematic analysis, and a combination of theoretical and sequential referral sampling [25,28-30] we might decide to interview representatives of public health agencies engaged in establishing influenza vaccination policies for healthcare organizations, representatives from the vaccine manufacturer, and hospital administrators.

Data collection and data analysis occur in conjunction with one another, in a recursive process [17,25,29,31,32]. Analysis of introductory interviews combines the review of filmed footage and written transcripts to identify codes, themes and concepts, as well as patterns of ideas that may serve as points of intersection for developing dialogue [15,17,29,31-33]. Comparisons within and across interviews are made (‘constant comparison’) [32-34]. A non-finalizing dialogic approach to analysis is adopted from this initial stage, whereby selections of the first interviews are compiled and compared, and points of divergence and convergence on the research topic identified [35]. The potential for a multiplicity of interpretations is recognized [35]. Based on this initial analysis, first ‘clips’^a^ of the individual interview films are assembled, highlighting each participant’s perspective, and how it may fit with others [2,35]. Interviewees participate with us in editing their own film clips to ensure:

(a) that they accurately reflect their perspectives; (b) that they reflect a balance between the general narrative of the participant’s perspective, and specific points of contention; and (c) that they feel comfortable showing them to others. This iterative editing process is the visual equivalent to the textual ‘report’ generation described by Frank in textual dialogic analysis, creating “an opening … to further representations” [35, p. 44].

Following the creation of each participant’s edited and distilled initial interview (the resulting “film clip”), it is shown to each of the other participants. Participants view each others’ clips individually, reflect on their meaning and implications, and frame their questions, comments and responses, which they later share with us on film. This ‘responsive interview’ session is also filmed, analysed, and then reviewed and edited with the participant’s input, until they feel comfortable showing the material to the other participants. This process is repeated a second time and a ‘second clip’ is assembled.

Finally, we review all approved footage and related transcripts. We go back and forth between the transcripts and the film clips. This allows us to examine the important narrative elements of the participants’ contributions textually within the transcripts and to analyse the degree to which the narrative lines we have identified as the core axes of the emerging dialogue actually ‘work’ on film. Subsequent analysis meetings involve sharing our views on how best to represent the dialogue based on the available data. We view preliminary assemblies of all the participants’ film clips and make decisions about which clips and which particular sequences contribute most to the clarity and coherence of the dialogue, and to the visual flow and pacing of the film, according to our analytic criteria (see below).

The ‘first cut’ assembles the final selections of clips from all the participants into a single dialogue. This cut of the film is shown to all participants individually for their review and approval. Participants offer further editorial feedback and can suggest edits to their own footage to ensure that they are comfortable with how they are portrayed in the resulting film. We incorporate this final round of feedback and make any necessary changes, taking care to preserve the integrity and meaning of the dialogue. This process can take several iterations (cut 2), and can require additional filming in some cases in order to clarify specific points or to fill logical gaps in the dialogue. Once all participants are satisfied with the final assembled film—the final cut or cut 3—we seek their permission to disseminate the film. Depending on the context and the sensitivity of the topic, we may seek permission for unrestricted dissemination, or for each specific instance of dissemination. *Brokered Dialogue* participants have been extremely supportive of this level of control over the representation of their own views, though it raises challenges in terms of the efficiency of production for the final films.

### Analysis

*Brokered Dialogue* is a qualitative research method with four specific analytic goals: 1) *where do participants start?* The goal is to understand differences and similarities in perspectives that participants bring to a given topic, i.e. their ‘baseline’ positions; 2) *where do they diverge?* The goal is to identify points of issue or contention within a chosen topic that can serve as moments of intersection among the participants’ perspectives and therefore also as key elements upon which to build a dialogue; 3) *how do they change?* The goal is to look for signs of evolution or change in individual participants’ perspectives that could be attributable to participating in the dialogue; and 4) *what are the pathways to progress*? The goal is to identify promising or novel pathways for progress towards solution or resolution of the issue under consideration that are revealed through dialogue.

Interviews are videotaped and audio taped to allow us to capture interactions between researchers and participants that occur off camera and incorporate them into the analysis. Audio tapes of all interviews are transcribed verbatim. We work from the text-based data to document emerging themes initially and to inform the editing process. We review the developing iterations of the film footage and use both written transcripts and the visual footage to inform the final rounds of edits.

Transcripts are reviewed and coded independently by all members of the project team. The dialogic narrative analysis is reviewed at team meetings and consensus reached. Editing decisions also incorporate the emerging analysis in order to organize the raw footage thematically as the resulting film is constructed. Accepted techniques for ensuring analytic rigour and trustworthiness for qualitative studies are employed, adopting a ‘relativist’ approach as characterized by Sparkes and Smith (2009) and others [3,36,37].

### Narrative analysis of textual data

We use narrative analysis to identify common themes and patterns in the written transcripts [31,33,34] and employ a combined approach of thematic, interactive and dialogic narrative strategies [27,35]. This approach analyses “how protagonists interpret things” [33, p. 5] and is concerned not only with the content of what participants say, but the form and tone of their accounts as well [5]. The experiences and perspectives offered by participants in the *Brokered Dialogue* (the protagonists) reveal important aspects of social life [34]. The interactive narrative approach [27] is particularly useful for studying the interactions of these perspectives, which is central to the *Brokered Dialogue* method. In particular, points of uptake and resistance to others’ perspectives are highlighted as potential moments of intersection of perspectives. A strategy of multiple readings of each transcript is adopted [35], with some readings undertaken to analyse the transcript as a story in its entirety, and others entailing line-by-line coding [32,34]. Constant comparison within and between transcripts is employed [38]. The embodied, emotional work of this kind of analysis is recognized [3].

In keeping with the narrative and dialogic narrative underpinnings of the method, we believe that judgments of inquiry quality should be grounded in Frank’s framework (2012), with its commitment to dialogic rather than monologic design (seeking multiple accounts from numerous tellers), a recognition of polyphony (interaction of each teller’s voice with specific others, both heard and anticipated), a recognition of the differing narrative resources at the disposal of differently positioned participants, and a recognition of the fluid and ongoing evolution of stories offered [2,35,39]. Along the same lines, *Brokered Dialogue* analysis is less about summarizing ‘findings’, and instead “aims at increasing people’s possibilities for hearing themselves and others” [35]. Prolonged and intensive engagement with participants is central and allows the researchers to interact with them and determine the degree to which they are comfortable with their accounts as represented on film and to gauge how their perspectives may have shifted over time. Quality is also assessed based on the coherence of the emerging film, the insights generated, and addressing the narrative tensions that emerge [40].We do employ techniques of constant comparative analysis, using multiple analysts, record keeping (of both data collection and analytic decisions), but always employing a narrative and non-finalizing lens throughout the project [35,37,38].

### Analytic approach to film editing

Analytic sessions focused on film editing are conducted in addition to the extensive analysis of textual data to determine which thematically-based segments should be incorporated into the final *Brokered Dialogue* films. Considerations of flow, pacing, timing and length constraints, visual and sound quality, and relative contribution to the resulting meta-narrative are incorporated into the crafting of the final film. The process is analogous to the writing up of qualitative research findings, marshalling evidence for themes through example quotes from participants. However the interaction of participants’ perspectives with one another is also portrayed in order to demonstrate the interests at stake in the topic, the resistance and uptake of other perspectives by particular participants.

The ‘brokering’ process is embedded within the method, as described above. However the core aspect of brokering is analytic – it is the analysts’ role to identify ‘points of intersection’ between particular participants’ accounts which contribute to ‘core axes’ for the dialogue. ‘Points of intersection’ occur within participants’ accounts, where they identify similarities between their perspective and those of other participants, or may emphasize a perspective differing significantly from those of other participants. Evidence of convergence and/or divergence of perspective are important to understanding the controversy and for illuminating possible pathways to solutions or resolutions. As analysts we then bring these convergences/divergences together on screen to make the key insights and opportunities for further dialogue explicit for the viewer. One further brokering role fulfilled by the researchers is to ensure respectful exchanges between participants, and to probe/challenge their positions on the topic at hand, in order to enhance clarity and to improve the efficiency of the dialogue.

### Research ethics considerations

Our *Brokered Dialogue* projects involve human research participants and therefore undergo ethics review by the St. Michael’s Hospital Research Ethics Board. The research ethics implications of conducting filmed interviews are considerable. All participants give written informed consent. All those invited to participate are told repeatedly that the nature of film means that they cannot be anonymous, and must agree to being filmed in order to participate. Consent is seen as an ongoing process, not a one-time event. Because they are recognizable on film, participants are reminded repeatedly that they have considerable editorial control over what gets shown to others. We use a participatory editing process. Because our goal is to promote in-depth and respectful interaction between key stakeholders with varying perspectives on a topic, we emphasize that they are not obliged to share any filmed segments that they would feel uncomfortable showing another person. They are invited to edit out any segments that they would not wish another person to see, or that they worry might be construed as offensive to other interested parties. No material is viewed by anyone other than members of the research team without the participants’ input and approval. Unlike documentary filmmaking, concerns of respect and abiding by participants’ wishes override those related to film quality. This core feature of *Brokered Dialogue* has been extremely well received by our participants.

### Limitations

While the method is innovative and has numerous strengths, it is not without its challenges. This technique presupposes a familiarity with qualitative narrative analysis and dialogic and interactive approaches to narrative analysis specifically [2,27,35] . For those less familiar with such approaches it may require a considerable investment of time and intellectual energy. In addition there are technical challenges inherent in working with filmmaking equipment, film editing, and visual data and analysis. We work extensively with experienced documentary filmmakers who are skilled in editing, directing, and camera work. We have found such interdisciplinary collaboration to be very rewarding. Finally, the technique itself, and particularly the participant-driven editing process can prove time consuming and labour-intensive. We intend to explore options for improving efficiency in future work.

## Summary

This paper has described the *Brokered Dialogue* method, a new approach we have developed to construct and analyse dialogue around controversial social issues. We have outlined the method’s theoretical foundations, its underlying assumptions, its key methodological features, and some reflections on potential uses and impact of the method. Two versions (a short 15-minute version and a longer 35-minute version) [41] of our pilot work, in which we studied a public drug funding policy controversy related to a diabetes drug are available for viewing via the following link: http://www.vimeo.com/brokereddialogue.

Our experiences in developing these pilot films have been our ‘laboratory’ for understanding and refining the core features of *Brokered Dialogue*. As such, the films do not fully reflect all the features of the method, as described above, but they provide a useful view of the origins of the method. Our current *Brokered Dialogue* projects better reflect the full scope of the method and these will be published and made available as they are completed.

We believe *Brokered Dialogue* is applicable in any context in which there is controversy, or where significant barriers to dialogue exist. It could be used to promote dialogue between homeless parents and children’s aid workers, between shelter users and neighbourhood residents, between patients and clinicians, and between injured workers and compensation board staff, or between researchers and policy makers, to name but a few examples. Our hope is that it will be used widely. This paper has served to introduce the method. In subsequent papers we will report on specific applications of *Brokered Dialogue* and also share our evolving views about how the method can be employed as an intervention strategy, in addition to its potential value as a research method.

## Endnotes

^a^A note on terminology: to clarify, we use the term ‘clip’ to denote film segments from individual participants’ interviews. ‘Cut’ refers to assemblies of footage that incorporate the perspectives of all the participants.

## Competing interests

The manuscript submitted does not contain information about medical devices or drugs. No benefits in any form have been, or will be, received from a commercial party related directly or indirectly to the subject of this manuscript.

## Authors’ contributions

Both JAP and JVL contributed equally to the conceptual and methodological development of this new method. JVL had the original idea for *Brokered Dialogue* and JAP provided important theoretical and methodological insights early on in its development. They have been research collaborators for the past five years. Both authors contributed equally to the subsequent data collection, data analysis and film editing during the study piloting the method and continue to do so in the subsequent projects where it is currently being applied. Both authors contributed equally to the preparation of this manuscript. Both authors read and approved the final manuscript.

## Pre-publication history

The pre-publication history for this paper can be accessed here:

http://www.biomedcentral.com/1471-2288/12/92/prepub
